# Feasibility and effectiveness of thoracic spine mobilization on sympathetic/parasympathetic balance in a healthy population - a randomized controlled double-blinded pilot study

**DOI:** 10.1186/s40945-019-0067-2

**Published:** 2019-12-09

**Authors:** Slavko Rogan, Jan Taeymans, Peter Clarys, Ron Clijsen, Amir Tal-Akabi

**Affiliations:** 1 Department of Health Professions, Discipline of Physiotherapy, Bern University of Applied Sciences, Bern, Switzerland; 2 Academy for integrative physiotherapy and training education, Grenzach-Wyhlen, Germany; 3 Vrije Universiteit Brussel, Faculty of Physical Education and Physiotherapy, Brussels, Belgium; 4 Rehabilitation Research Laboratory, Department of Business Economics, Health and Social Care, University of Applied Sciences and Arts of Southern Switzerland, Landquart, Switzerland

**Keywords:** Autonomic nervous system, Musculoskeletal manipulations, Heart rate variability

## Abstract

**Background:**

Physiotherapists often use thoracic spine mobilization (TSM) to reduce pain in patients with back disorders via a reduction of sympathetic activity. There is a “trade-off” in the activity of the sympathetic and parasympathetic nervous system activity. A sympathetic/parasympathetic balance (SPB) is needed to guarantee body homeostasis. However, body homeostasis is seldom considered as an aim of the treatment from the perspective of most physiotherapists. Strong empirical evidence for the effects of TSM on the SPB is still lacking.

Some studies showed that spinal manipulation may yield beneficial effects on SPB. Therefore, it could be hypothesized that TSM is feasible and could influence SPB reactions. The primary aim was to describe the participants’ adherence to the intervention and to the measurement protocol, to identify unexpected adverse events (UAE) after TSM, to evaluate the best method to measure SPB parameters (heart rate variability (HRV), blood pressure (BP), heart rate (HR), skin perfusion and erythema) and to estimate the investigation procedure. The secondary aim was to assess the effects of TSM on SPB parameters in a small sample of healthy participants.

**Methods:**

This crossover pilot study investigated TSM using posterior-anterior mobilization (PAM) and anterior-posterior mobilization (APM) on segments T6 to T12 in twelve healthy participants during two consecutive days. To evaluate feasibility, the following outcomes were assessed: adherence, UAE, data collection and data analysis. To evaluate the effect of TSM on SPB, HRV, BP, HR, skin perfusion and erythema were measured.

**Results:**

The adherence was 100%. No UAE were reported. PAM showed larger effect sizes compared to APM in many secondary variables.

**Conclusions:**

Although 100% maximal adherence was reached and no UAE were observed, data recording in future studies should be done during a second time interval while the data transfer from device to the computer software should occur immediately after completion of each participant’s measurement. The results of this pilot study suggest that PAM can reduce HRV HF and HRV ratio LF/HF and increase HR.

**Trial registration:**

ClinicalTrail.gov (NCT02832141).

## Background

In the literature the parasympathetic nervous system (PNS) and the sympathetic nervous systems (SNS) have been described in two different anatomical and functional areas [1–5]. The function of the PNS consists of conservation and restoration of energy supply by causing a reduction of heart rate (HR) and blood pressure (BP). In contrast to the PNS, the SNS increases HR, BP and causes a diversion of blood flow from the skin, the gut and splanchnic vessels to those supplying skeletal muscle during physical activity. Under normal condition (healthy state), a fine balance between PNS activity and SNS activity exists to maintain homeostasis. This sympathetic/parasympathetic balance (SPB) can be disturbed by PNS withdrawal and SNS overactivity due to a variety of factors such as changes in internal and external environment.

Many studies have evaluated the effectiveness of spinal manual therapy intervention on the SNS. For example, Slater et al. [6] found that the novel manual therapy technique (MTT) called “sympathetic slump” applied on 22 healthy persons influenced peripheral SNS activity. Zegarra-Parodi et al. [7] observed an increased peripheral skin blood flow, indicating a decreased SNS activity, after spine mobilization in 32 healthy volunteers. Vincenzino et al. [8] and McGuiness et al. [9] could demonstrate that cervical spine mobilization techniques significantly increased HR and systolic BP. In contrast, Sampath et al. [10] found no short-term changes in SNS activity in 24 healthy men following thoracic spinal manipulation.

However, studies investigating MTT on SPB are very scarce. In clinical and research settings in physiotherapy, the SNS is largely included in explanatory models for the development and chronification of pain. In osteopathy, for example, a model hypothesizes that disorders of the SNS can promote and intensify disease processes. Especially the SNS can influence the trophicity of various tissues as well as the blood circulation of the skin in the healthy and patients. The SNS with its paravertebral ganglia, which are adjacent to the thoracic spinal column, is regarded as a body area that could stimulate by MTT of the vertebrae [11].

Several measurement methods have been developed to assess SPB. These include heart rate variability (HRV), blood pressure (BP), heart rate (HR), and for SNS activity, skin blood flow and erythema measurements. For example, HRV and systolic BP have been described as valid (indirect) methods to assess SPB [12, 13]. Frequency-specific fluctuations in HR can be evaluated with power spectrum analysis of HRV [14]. High frequency (HF) components between 0.014 and 0.40 Hz reflect the activity of the vagal tone (PNS). Low frequency (LF) components between 0.04 and 0.15 Hz reflect activity of SNS while the HRV ratio LF/HF reflects SPB [15].

New findings from the neurophysiological research are increasingly being transferred into manual therapy. MTT may yield effects via two types of pathways [16]: a biomechanical pathway focusing on improved joint mobility (arthrokinematics) and a multidimensional physiological pathway. The latter involves mechano-cellular signal transduction leading to cellular responses. Furthermore, reflex modifications which have been described as the activation of different reflex loops via the peripheral nervous system (NS), the SNS and PNS after spinal manual therapy. Several studies have described the effects of spinal manipulation on HRV [13, 17, 18]. In contrast, there is little empirical evidence on the effects of thoracic spine mobilization (TSM) on SPB. There are currently three studies that have investigated the effects of TSM on the SPB [11, 19, 20]. A single case study [21] observed that TSM in a young male volunteer resulted in a decreased skin perfusion and erythema in the thoracic segment, suggesting an acute increase of SNS activity. One pilot study found that posterior-anterior mobilization (PAM) on T1 to T5 segments of eight healthy, young participants had a tendency to reduce HR while no effects on HRV and BP were observed [20]. The authors of one study [11] concluded that grade III posterior-anterior rotatory mobilization techniques applied on the thoracic vertebra T4 can produce sympatho-exitatory effects in 36 healthy volunteers.

Randomized controlled trial (RCT) is a strong study design to draw conclusions on causality [22, 23]. However, RCT is often resources and time consuming and therefore its application requires careful consideration. Eldridge et al. [24], Rogan and Karstens [25] and Thabane et al. [26] described the purposes of pilot and feasibility studies. These authors explained that the term “pilot study” is commonly used to evaluate feasibility of the methods for a planned larger RCT to guarantee a valid study design and an appropriate sample size.

To date, only one pilot study on the possible effects of TSM (T1 to T5) on HRV, BP and HR as proxies for SPB has been published [20].

The primary aim was to evaluate the feasibility of the methods and the study protocol measured as participants’ adherence, UAE, data collection and data analysis (i.e. identifying the best methods to measure HRV, BP, HR, segmental skin perfusion and erythema). The secondary aim was to assess possible effect sizes of TSM (T6 – T12) on HRV, BP, HR, segmental skin perfusion and erythema as proxies of SPB in healthy persons.

The results may be important for the accurate planning of future larger RCT studies (e.g. for more adequate sample size calculation).

## Methods

### Study design

**Fig. 1:**
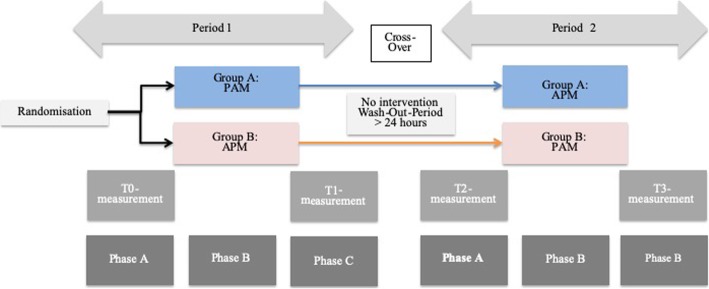
Flow Chart

This pilot study included twelve healthy adults (aged from 20 to 30 years) who were randomly allocated into two similar independent groups, with two interventions and in two separate sessions (Fig. 1). The randomization of the participants was performed by an blinded statistician. Concealed allocation was performed using an opaque sealed envelope method. This study was registered at ClinicalTrail.gov (NCT02832141) and was approved by the local ethic committee in Bern, Switzerland (KEK-2016/00320). All included persons were free of any acute physical complaints during the previous four weeks. Exclusion criteria were chronic pain, osteoporosis, blood pressure medication, acute infections, fever, fractures of the spine or pelvis (< 12 months), cardiac disease, neurological disease, peripheral vascular disease, thrombosis and pregnancy. The participants provided written informed consent before inclusion.

### Study procedure and protocol

Figure 1 describes the flow of the study.

Group 1: During session 1 (day one) and in prone position, a grade 3 oscillatory mobilization in a rhythmical fashion (120 movements per minute) [27] of the thoracic spine with PAM was applied for 100 s on every thoracic segment, from T6 to T12. A wash-out phase of at least 24 h was guaranteed [19, 20]. During session 2 (day two) and in sitting position, a grade 3 oscillatory mobilization in a rhythmical fashion (120 movements per minute) but now with anterior-posterior mobilization (APM) was applied for 100 s on every thoracic segment from T6 to T12.

Group 2: During session 1 (day one) and in sitting position, a grade 3 oscillatory mobilization in a rhythmical fashion (120 movements per minute) with APM was applied over 100 s on every thoracic segment from T6 to T12. A wash-out phase of at least 24 h was guaranteed. During session 2 (day two) and in prone position, a grade 3 oscillatory mobilization in a rhythmical fashion (120 movements per minute) of the thoracic spine with PAM was applied over 100 s on every thoracic segment from T6 to T12.

Each session was divided into three phases (A, B, and C) (Fig. 1). The investigation took place in a closed room with little noise disturbance [28] to ensure a comfortable atmosphere at a constant temperature of 23 °C.

**Fig. 2:**
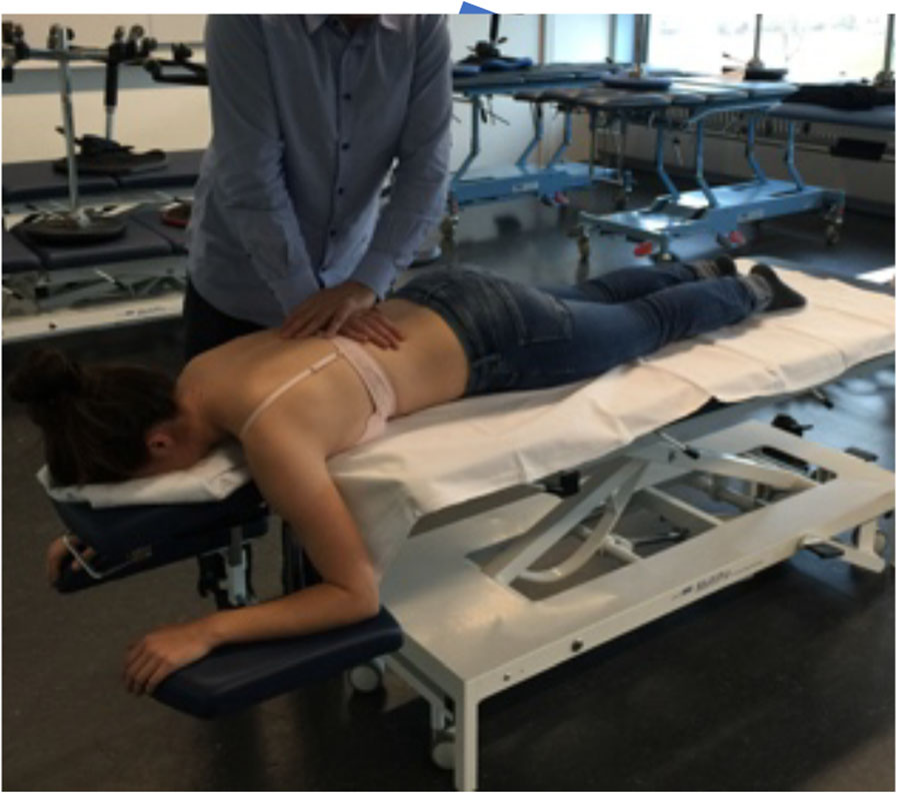
Posterior-anterior mobilization

Phase A was the same for both groups. It started with a resting, familiarization period (ten minutes) to allow stabilization of BP and HR. HRV, systolic BP (sBP) and diastolic BP (dBP), HR, perfusion of the superficial skin plexus and erythema were assessed after eight and ten minutes by two blinded assessors (blinded towards the intervention). These assessors were trained before the start of the study. During phase B the intervention (PAM and APM) was applied. PAM was applied with the participant lying in prone position with a towel placed under the head to ensure a horizontal and comfortable neck position (Fig. 2).

**Fig. 3:**
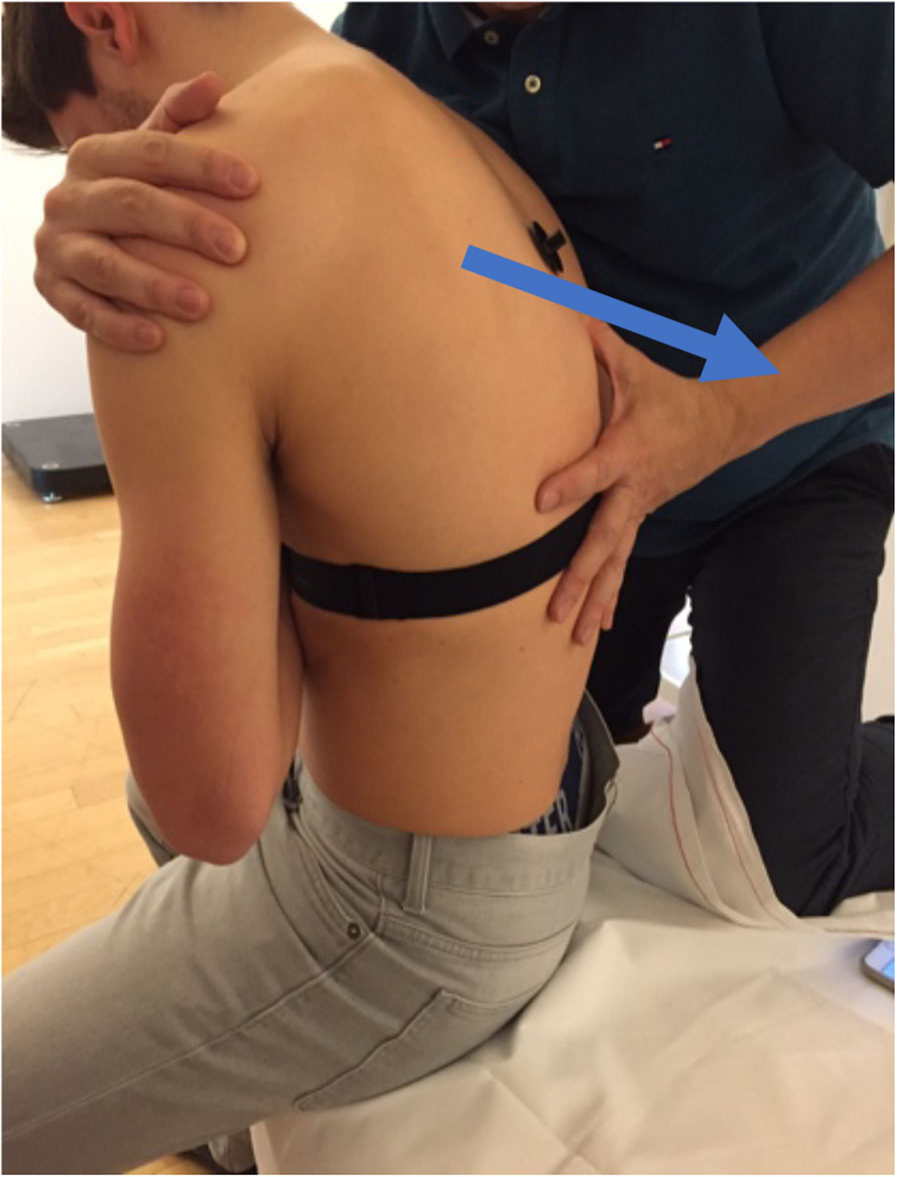
Anterior-posterior mobilization

The physiotherapist applied a vertical pressure on the processus spinosus with his hands to exert large amplitude movement that reaches the end range of movement. APM was carried out in sitting position with kyphotic posture [20]. The caudal processus spinosus of the vertebra to be mobilized was fixed with the left thumb of the physiotherapist (Fig. 3) who applied posterior pressure with the right hand and the right shoulder.

Phase C was another resting period during which HRV, sBP, dBP, HR, skin perfusion and erythema were measured immediately and after two, four, six, eight, and ten minutes by two blinded assessors. For the analysis all measurement time points were averaged.

To identify the starting T6 level of the thoracic spine, each participant was assessed by the same experienced physiotherapist. First, C6 was identified by palpating the lowest freely moving spinous process as compared to the following static spinous process C7 [29]. Second, every spinous process from C7 was palpated caudally towards the T6 level. The passive intervertebral motion test was carried out to identify T6 [29]. A clear marker was placed on the skin identifying T6. Participants should not wash the marker place on the skin with soap the ensure visibility of the marker place the next day.

### Feasibility outcome variables

Success criteria for feasibility were defined as: 1) adherence: drop-out of 0% and a 100% presence based on a previous feasibility study [30], which considered these cut-offs as acceptable adherence values for acute effects measurements. 2) UAE after TSM were recorded following the criteria as proposed by Puentedura and O’Grady [31] UAE with moderate to severe symptoms leading to a serious, distressing and unacceptable state of the participant (e.g. pain, headache, fatigue) and requiring further treatment should not occur (cut-off 0%). 3) Feasibility of measurement methods was determined by successful data recording (100%), percentage of missing data (less than 5%), and the time management of the investigation procedure (less than ten minutes).

### Physiological outcome variables

The HRV parameters were recorded and stored using a Polar heart rate monitor system (Polar Electro Oy, Kempele, Finnland). The breast belt (two-leads) measured R-R intervals at 1000 Hz frequency and transmitted the R-R interval values (via the W.I.N.D.-transmitter) to the wristwatch (RS800CX), where data were stored.

R-R intervals are the intervals in time between consecutive heart beats, i.e. between two consecutive R peaks in the ECG curve.

Criterion validity (r = 0.99) and the instrument reliability were found to be excellent in the Polar® RS800CX heart rate measuring system [32]. Low frequency (LF) and high frequency (HF) band and LF-to-HF band ratio values were used to evaluate HRV. HRV indicates the beat-to-beat variation and can be seen as a proxy of SPB.

BP was measured using the Tensoval Duo Control (Paul Hartmann AG, Heidenheim, Germany) upper arm blood pressure monitor. This system can be recommended for clinical and home care use in an adult population according to the criteria of the British Hypertension Society protocol [33]. The reference BP was measured according to Riva-Rocchi [34] and expressed in mmHg (millimeters of mercury) for sBP and dBP values.

HR was recorded using the Polar (RS800CX) system. The values of HR measurement were averaged over a period of one second.

**Fig. 4:**
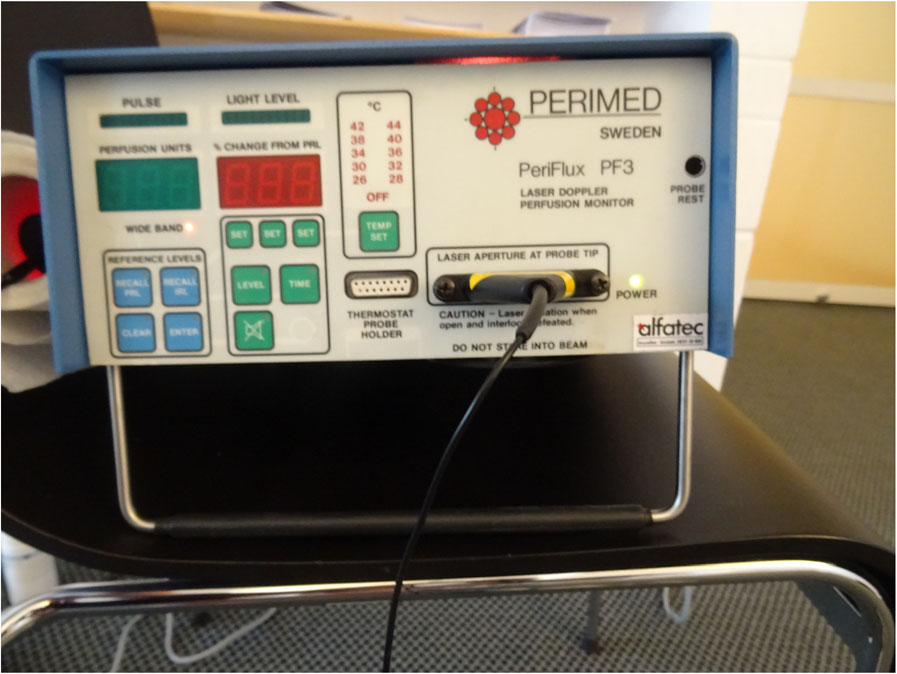
PeriFlux 4001 Master

Perfusion of the thoracic skin plexus at T6 level was assessed using the PeriFlux 4001 Master (Perimed AB, Järfälla, Sweden) (Fig. 4). The monochromatic, coherent laser light of the Laser Doppler Flowmeter (PeriFlux) is scattered by the erythrocytes moving in the blood vessels and thrown back. The reflected laser light is detected photoelectrically and compared with the output value of the Doppler frequency. An unpublished reliability study previously conducted by the authors showed a good intra-rater reliability (rho = 0.924; *p* = 0.01), a moderate inter-rater reliability (rho = 0.615; *p* = 0.05) and a good test-retest reliability of (rho = 0.738; *p* = 0.01). The values for perfusion of the skin microcirculation were obtained as arbitrary units (A.U.) [35].

**Fig. 5:**
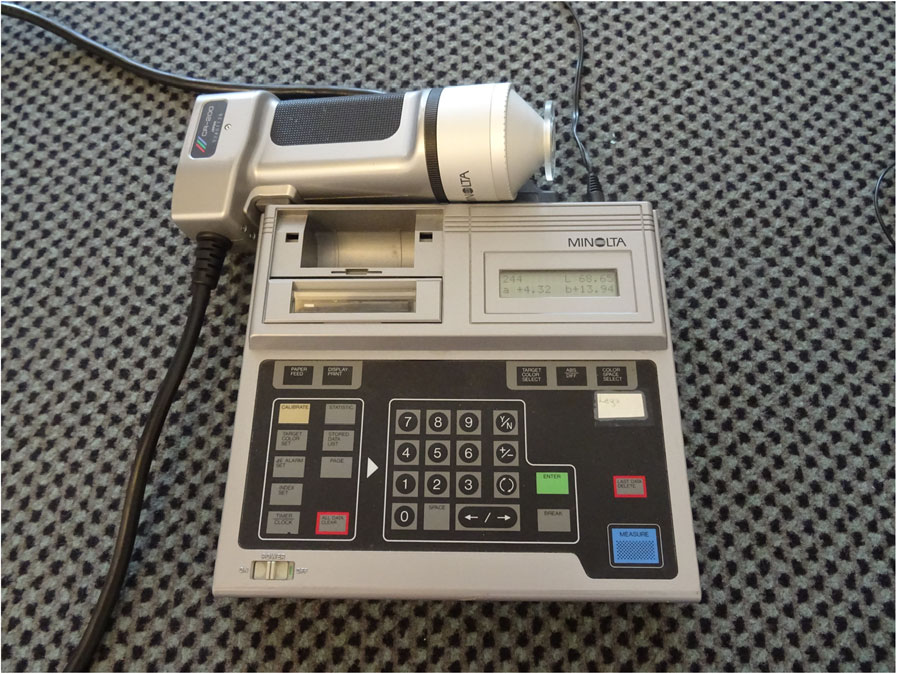
Chromameter

Erythema of the thoracic skin plexus at T6 level was evaluated by tristimulus surface colorimetry using a Minolta CR 200 Chromameter (Minolta, Marunouchi, Japan) (Fig. 5). To evaluate the change in skin color, the colorimetric measurements were performed with the chromameter operating in the International Commission on Illumination L*a*b* color space. An unpublished reliability study previously conducted by the authors showed a very good intra-reliability (rho = 0.964; *p* = 0.01), a very good inter-rater reliability (rho = 0.974; p = 0.01) and a very good test-retest reliability (rho = 0.978; p = 0.01). To quantify the change of skin-surface color and erythema, the a* parameter is used, as it represents the chromaticity between red/magenta and green (negative values indicate green, while positive values indicate magenta).

### Data transfer and analysis

HRV and HR data measured from the Polar heart rate monitor and stored to the watch were transferred to the Polar Pro Trainer 5 software program by two blinded assessors using infrared sensors. Data were saved on the PC for further analysis at the end of day one and day two. The assessors were blinded towards the TSM. The blood pressure values, the skin perfusion values, and the erythema values were written on a case report form during the intervention. After the intervention period two blinded assessors inputted these values to an excel sheet. One assessor did the imputation while the other assessor double-checked the data for reasons of quality control.

The analysis of all outcome data was conducted by an independent (blinded) statistician once all measurements were finalized.

### Statistics

Participants characteristics were summarized using means and standard deviations for descriptive statistics. This current pilot study with a 2 × 2 design used a linear model for nonparametric analysis to present the effect sizes [36] because of the difficulties in verifying normality with a small sample size (*n* = 12).

The effectiveness of PAM and APM was assessed on the basis of the within-subject differences between the two mobilization techniques with regard to the outcome variables [37]. This study only analyzed data acquired after intervention in period 1 (T1) and period 2 (T3) [36, 37]. The present study used a confirmatory data analysis to check carry-over effects, periodic effects and intervention effects. The impacts of the first intervention should not be present when participants enter the second intervention period. The mean of the sum of each variable under investigation (e.g. HR) of period one and period two in group one and the sum of period one and period two in group two was compared to assess this carry-over effect [36]. In this case, the within-subject sums of the outcomes from both periods were compared by a Wilcoxon rank sum test. When carry-over effects were observed, only the first period was calculated. When there were no carryover effects, periodic effects and treatment effects could be analyzed. The period effect was evaluated by comparing the difference between APM technique and PAM technique for group 1 and group 2. Mean differences for group 1were calculated after time period 1 minus after time period 2. Mean differences for group 2 was calculated after period 1 minus after period 2. To analyze periodic effects a Wilcoxon rank sum test was used [36, 37]. The effectiveness of TSM was evaluated by comparing the within-subject differences between period 1 and period 2. For group 1, the within-subject differences were calculated after period 1 minus after period 2 and for group 2 the within-subject differences were calculated after period 2 minus period 1 using the Wilcoxon rank sum test.

Results were standardized to Z-scores and the formula r = Z /√n was used to calculate the effect sizes. The following benchmarking for the interpretation of the effects size was followed: r around 0.1 (small effect), r around 0.3 (medium effect) and r above 0.5 (large effect) [38].

## Results

A total of twelve participants volunteered in this pilot study (Table 1).

**Table 1:** Demographic and anthropometric data in means and standard deviation (±)

Sex	n	Age (years) mean (±)	Height (cm) mean (±)	Weight (kg) mean (±)
Men	5	24.80 (± 3.96)	185.20 (± 5.89)	86.60 (± 10.97)
Women	7	22.29 (± 1.25)	169.86 (± 6.36)	69.14 (± 8.28)

Legend: *N* number of participants, *cm* centimeter, *kg* kilogram

### Feasibility outcomes

Adherence was 100% while no UAE were observed. Correct calculation of the HRV was hampered because of a limited storage capacity of the Polar RS800CX watch. It was found that the Polar wristwatch could not store all data of all volunteers and automatically saved only the 5 s R-R interval data instead of the needed 1 s R-R- data. The planned time to conduct the measurements (ten minutes) was found to be adequate.

### Physiological outcomes

No carry-over effects were observed.

Table 2 represents the results of the physiological outcomes.

**Table 2:** Results of physiological intervention effects and periodic effects. Values are given in median and interquartile range, as *p*-value and effect sizes (r)

	Period 1	Period 2	Intervention effect	p / r	Period effect	p / r
Group 1: HRV HF	907 (422–1891)	1567 (789–1893)	− 172 (− 1290–266)	0.025* 0.65	−172 (− 1290–266)	0.109 0.46
Group 2: HRV HF	2627 (834–6801)	950 (429–2699)	1237 (237–3555)		236 (1237–3556)	
Group 1: HRV ratio LF/HF	94 (75–273)	88 (67–113)	29 (− 12–155)	0.016* 0.70	29 (−12–155)	0.423 0.23
Group 2: HRV ratio LF/HF	52 (30–109)	156 (36–209)	−86 (− 109 - -12)		−1237 (−3555 - -237)	
Group 1: HR	61.25 (58.00–70.21)	59 (52.25–68.23)	4.78 (3.32–8.15)	0.006* 0.78	4.78 (3.32–8.15)	0.423 0.23
Group 2: HR	53.50 (52.62–62.75)	58.14 (54.11–63.89)	- 3.19 (−6.02–1.28)		3.19 (−1.28–6.02)	

Legend: *HRV- HF* heart rate variability high frequency, *HRV ratio LF/HF* heart rate variability ratio low frequency/high frequency, *HR* heart rate frequency

* significant < 0.05

PAM in prone position yielded statistically significant decrease on HRV HF (r = 0.65; *p* = 0.025) and HRV ratio LF/HF (r = 0.70; *p* = 0.016) and significant increase on HR (r = 0.78; *p* = 0.006).

## Discussion

This pilot study was conducted in order to evaluate the feasibility of the study design using three specific criteria of success and possible effects of TSM (T6 – T12) on HRV, BP, HR, segmental skin perfusion and erythema as proxies of SPB.

The success criteria set a priori for adherence (0% drop-out and a 100% presence of participants). The absence of moderate or severe UAE as well as the time need to conduct the measurements (less than 10 min) could be fulfilled.

The compliance to the TSM (T6 – T12) intervention was good. All 12 participants completed all the intervention sessions. These results are not in line with the study from Tal et al. [20] observed an adherence rate of 75%. It remains difficult to give a clear-cut explication to this observation.

Safety was an important outcome at the current study. UAE were defined based on Puentedura and O’Grady [31] who evaluated UAE after thrust joint manipulation. The authors concluded that after such thrust manipulation UAE of the thoracic spine might occur, most often as trauma to the spinal cord followed by pneumothorax. They concluded also that thrust manipulation of the spine differs significantly from other manipulations. Therefore, the absence of UAE observed in the present pilot study may be at least partially be explained by the use of TSM which is applied with less velocity and lower force to the spine as compared to thrust manipulation.

When planning future studies in this field, an adjustment in the measurement procedure of R-R data-gathering is needed. To the best of our knowledge, the user manual of the Polar system does not describe the possible limited storage capacity of the Polar watch. Another possibility could be to use 2 Polar V800 watches and to upload the recorded data in the Polar flow and to perform the R-R data analysis in the Kubios software afterwards.

Therefore, the researchers in this present pilot study assumed that data from all volunteers could be stored at once for an R-R one-second-interval analysis. When planning a new study with Polar, the protocol should consider an R-R data transfer from the Polar wristwatch to the PC immediately after each participant’s R-R registration. In addition, researchers should immediately conduct the quality of the R-R data on the PC to ensure proper 1 s HRV afterwards. HRV can also be measured using electrocardiography (ECG). The transmission of cardiac activity takes place via the measuring electrodes via a cable connection to the ECG device. This current pilot study followed the recommendations of prior articles [24–26] and evaluated the feasibility of the methods of this crossover pilot study while objectively declaring the results as preliminary.

### Preliminary estimates of TSM

The secondary aim of this current pilot study was to estimate potential effects of TSM (T6 – T12) on SPB in healthy volunteers. The results of the present pilot study suggest that TSM (T6 - T12) using PAM but not APM has the ability to induce SNS and PNS stimulation. The target parameters HRV HF and HRV ratio LF/HF and HR, changed significantly with large effect sizes after PAM. HF is related to activity of the PNS. HR frequencies and HRV ratio LF/HF are related to specific SNS activities. The HR frequency increases during PAM which is more likely to increase SNS activity. The findings of the present pilot study corroborate previous results by Budgell and Polus [39]. They examined 28 healthy young adults between the ages of 18 and 45 years in their crossover study and found significant changes after thoracic spine manipulation in HF (*p* = 0.004), in LF (*p* = 0.020) and in the HRV ratio LF/HF (*p* = 0.003). The authors explained the changes in HRV as a result of the thrust manipulation at the end of the expiratory phase with an open glottis. They assumed that in prone position the mechanoreceptors in the lungs are discharged. Furthermore, TSM may cause an increase in chest pressure. This mechanical component may influence the functioning of the heart and its surrounding large vessels which increases blood pressure and activates the mechanoreceptors in the cardiovascular system.

The PAM in the present study was performed in prone position. As already mentioned above [39], mobilization and manipulation in prone position lead to an increase in chest pressure resulting in a discharge of the mechanoreceptors of the lungs. The APM in the present study was performed in a sitting position with a kyphotic thoracic spine position. The mechanical component, as described by Budgell and Polus [39], is omitted in the sitting position. For the current study, the mechanical pressure component cannot be considered as justification of the HRV changes.

However, this study differs from the findings of Sampath et al. [10] who demonstrated that supine thoracic spine manipulation showed no effects on the SPB. They pointed out that the initial prone and supine positions have an influence on HRV.

The results of the current study suggest that the HR increases significantly after PAM compared to APM. Jowsey and Perry [11] could demonstrate that TSM in rotation significantly increased sympathetic activity in the hands of healthy participants. They applied a grade III postero-anterior rotatory joint mobilization techniques to the T4 at a frequency of 0.5 Hz.

On the other hand, Tal et al. [20] found a significant HR reduction (r = 0.75; *p* < 0.03) after PAM in healthy participants, applied at T4. Kingston et al. [40] described positive changes of the HR in their systematic review after spine mobilization in healthy participants.

Following a “sympathetic slump mobilization” Slater et al. [6] found a decreased skin temperature indicating an increase in vasomotor activity. The latter may be associated with an increased SNS activity. The present study observed, however, no changes in skin perfusion and erythema. The discrepancy of the results between Slater et al. [6] and the current study could be due to different methods of measurements (AT64 Portables Single-Channel SCR device versus Laser Doppler Flowmetry). According to Slater et al. [6], a Laser Doppler Flowmetry may represent a more sensitive method for detecting changes in vasomotor function.

### Limitation of the study

This current pilot study involved healthy participants. Healthy participants are included in research, when there is insufficient clinical research or insufficient data on a particular topic [41]. Caution is warranted, how patients will respond in a clinical setting. Studies with healthy participants can help clinicians understand what is normal and might lead to hypotheses concerning best treat for persons with a disease or injury condition. Reis et al. [42] investigated the effects of Maitland mobilization grade III posteoanterior central pressure glide in patient with Fibromyalgia and healthy persons. Fibromyalgia patients showed reduced HRV compared to healthy persons.

The standardized prone position was used for the measurements and for PAM, while TSM with APM direction was carried out in sitting position. It is known that HRV LF value elevated under the following criteria: 90-degree tilt, standing, mental stress, performing moderate exercise, physical activity, occlusion of the coronary arteries or carotid arteries, generally during the day. In this context, the sitting position can be considered as very similar to that of the standing position. Even the intervention time and not the posture could have influenced the outcome variable HRV LF.

In short-term regulation, the sympathetic outflow is regulated by homeostatic feedback mechanisms via the arterial baroreflex [43]. Blood flow measured as perfusion of the superficial skin plexus and the change in skin color as erythema. Laser Doppler flowmetry (LDF) is an established and commonly used, non-invasive method to assess perfusion of the skin microcirculation. At this study perfusion and erythema showed opposite reactions but were stable after TSM.

Some limitations can be addressed to the use LDF in this setting. Compared to a speckle pattern which is a random granular interference pattern produced by (laser) light reflected or scattered from different parts of the illuminated surface. The laser speckle contrasting imaging (LSCI) allows full-field imaging of skin perfusion in near real time due to faster signal processing [44]. LSCI could measure a larger skin area compared to the spot wise 3 mm measurement of the LDF. Therefore, for the upcoming study it should be considered to perform the measurement of the skin microcirculation with a LSCI system.

Furthermore, a localization of and intervention on a specific thoracic level is not always simple. There is evidence that reliability and validity of palpation techniques for identifying spinosus processus are not precise enough [45]. Furthermore, as neighboring joints of the spinal column are also move during continuous mobilization, isolated segmental mobilizations cannot be performed reliably on the spine.

Another limitation is the unpublished reliability data of the PeriFlux 4001 Master (Perimed AB, Järfälla, Sweden) and the Chromameter (Minolta, Marunouchi, Japan).

## Conclusion

A maximal adherence was reached (100%). Both TSM techniques were safe and did not produce any UAE. Data recording in future studies should be done during a second time interval while the data transfer from wristwatch to the computer software should occur immediately after completion of each participant’s measurement. The findings of this current study suggest that PAM can reduce HRV HF and HRV ratio LF/HF and increase HR.

## Data Availability

The datasets used in this current study are available from the corresponding author on reasonable request.

## Abbreviations

ANS: Autonomic nervous system
APM: Anterior-posterior mobilization
BP: Blood pressure
cm: Centimeter
DBP: Diastolic blood pressure
HF: High frequency
HR: Heart rate
HRV: Heart rate variability
kg: Kilogram
LDF: Laser Doppler flowmetry
LF: Low frequency
LSCI: Laser Speckle contrast imaging
mmHg: Millimeters of mercury
PAG: Periaqueductal gray
PAM: Posterior-anterior mobilization
PNS: Parasympathetic nervous systems
RCT: Randomized control trial
RR: Riva-Rocci
SBP: Systolic blood pressure
SNS: Sympathetic nervous systems
TSM: Thoracic spine mobilization
